# Concerns about covert HIV testing are associated with delayed presentation in Ethiopian adults with suspected malaria: a cross-sectional study

**DOI:** 10.1186/s12889-016-2773-y

**Published:** 2016-02-01

**Authors:** Frew Tadesse, Wakgari Deressa, Andrew W. Fogarty

**Affiliations:** 1 School of Public Health, College of Health Sciences and Medicine, Jigjiga University, Jigjiga, Ethiopia; 2 Department of Preventive Medicine, School of Public Health, College of Health Sciences, Addis Ababa University, Addis Ababa, Ethiopia; 3 Division of Epidemiology and Public Health, University of Nottingham, Nottingham, UK

**Keywords:** Malaria, HIV, Concern, Diagnosis, Delay, Stigma, Ethiopia

## Abstract

**Background:**

Although early diagnosis and prompt treatment is important in preventing mortality from malaria, presentation of symptomatic individuals is often relatively late. One possible contributing factor is that fear of covert human immunodeficiency virus (HIV) testing delays presentation in adults. We aimed to survey the magnitude of such concerns and their association with delayed presentation with suspected malaria.

**Methods:**

The study design was a health facility-based cross-sectional survey. The study population consisted of adults with suspected malaria who presented to health centres in central Ethiopia. Data were collected on attitudes to HIV testing and the duration between onset of symptoms and treatment seeking for suspected malaria.

**Results:**

Eight hundred and ten individuals provided data. Of these, 406 (50 %) perceived that HIV testing was routinely done on blood donated for malaria diagnosis, and 327 (40 %) considered that community members delayed seeking medical advice because of these concerns. Concerns about HIV testing were associated with delays in attending for malaria diagnosis and treatment, with 117 individuals (29 %) of those with concerns about covert HIV testing waiting for 4 days or more, compared to 89 (22 %) of those who did not have any such concerns (*p* = 0.03). One hundred and twenty nine (16 %) individuals stated that concern about HIV testing was the main reason for the delay in seeking treatment, and 46 % of these individuals presented after experiencing symptoms of malaria infection for three days or more compared to 22 % of the 681 individuals who had no such concerns (*p* < 0.001). Analysis stratified by health centre demonstrated that these associations were a consequence of Meki health centre (odds ratio for duration of symptoms greater than 3 days if patient has concerns about HIV testing was 8.72; 95 % confidence intervals 3.63 to 20.97).

**Conclusions:**

In adults living in central Ethiopia, the perception that HIV testing accompanied the investigation of suspected malaria was common. This is likely to impede presentation for early medical treatment in some areas and represents a reversible risk factor that deserves further study.

## Background

Malaria is an important treatable cause of morbidity and mortality, with 3.4 billion people globally estimated to be at risk of infection in 2012, resulting in approximately 200 million cases of malarial infection and 600,000 deaths annually [1]. In Ethiopia, malaria is one of the most important public health problems, representing the commonest communicable disease [2] and imposing a high economic cost on the country [3]. As malaria is treatable with prompt medical care [4], the national malaria programme stresses the importance of early treatment-seeking behaviour for suspected infection [2].

Ethiopia is one of the countries in the sub-Saharan Africa that have a generalized human immunodeficiency virus (HIV) epidemic with about 750,000 people living with HIV/AIDS [5]. The prevalence of HIV infection among adults aged 15–49 years old in 2011 was estimated to be 4.2 % in urban areas and 0.6 % in rural areas [6]. Despite the availability of anti-retroviral treatment, individuals may be reluctant to undergo testing of their HIV status, as they do not wish to be exposed to the knowledge and social consequences of a potential positive diagnosis.

We recently reported data demonstrating that presentation of children with suspected malaria was delayed if their guardian were concerned that blood samples collected for malaria diagnosis were being covertly tested for HIV status without consent [7]. A similar hypothesis was tested in adults, speculating that concerns about covert HIV testing were common and resulted in delay in the presentation of symptomatic patients for investigation of malaria. This hypothesis was tested using a cross-sectional observational study design in Ethiopia in adults with suspected malaria visiting health centres.

## Methods

### Study population

The study design was a health facility-based cross-sectional survey. This study was conducted in East Shewa Zone of Oromia Regional State in Ethiopia, where malaria is one of the leading health problems. Five health centres were included in the study and data were collected in October and November 2012, which represents the peak malaria transmission season in this part of Ethiopia. Each health centre is led by medically qualified clinicians, has diagnostic facilities for malaria and covers a population of approximately 20,000 individuals. All adults aged 16 years or over who presented with symptoms consistent with malaria and gave finger-prick blood for microscopic examination at the health centres were eligible to participate in the study. All participants gave informed consent and received standard treatment for their presenting condition.

### Sample size calculation

The aim was to recruit 850 individuals. The total sample size allocated to each health centre was proportional to the total patients tested for malaria in the previous 3 months (June-August, 2012) and individuals were recruited consecutively until the estimated sample size was achieved. Assuming that 20 % of patients had concerns about HIV testing in population and 50 % have had symptoms for 5 days or less, it was estimated that the study would have over 90 % power (5 % risk of type I error) to detect an absolute increase of 20 % in patients who had concerns about HIV testing who experienced malaria symptoms for 5 days or more compared to those who had had no such concerns.

### Data collection

A pre-tested structured questionnaire was initially developed in English and then back translated into local language (*Afan Oromo*) and used for data collection. The questionnaire collected data on socio-demographic characteristics, concerns about HIV testing and duration of symptoms. One data collector and one supervisor from each health centre were trained to recruit the study participants and implement the questionnaire. Subsequent diagnosis of malaria was done using blood film examination under microscopy.

### Statistical analysis

The main exposure of interest was the response to the question ‘*Are you concerned about HIV testing when you visit a health facility for malaria diagnosis and treatment?*’ All completed data were examined for completeness and consistency. Initial analysis used Mann–Whitney and Kruskal-Wallis tests to identify differences in the duration of symptoms between different measures of concerns about HIV testing and perceptions of these concerns in the wider community. As the differences observed were relatively small, with similar median values as a consequence of the structure of the data, the outcome variable of duration of time was categorized into either less than or equal to three days and more than three days. Statistical analysis used chi-squared testing and subsequent analysis by logistic regression stratified by health centre. The impact of urban and rural locations on the primary exposure was explored as the prevalence of HIV infection varied between these settings.

### Ethical considerations

The study protocol was reviewed and approved by the Research and Ethics Committee of the School of Public Health at the College of Health Sciences. Informed consent was obtained from each participant and confidentiality was maintained. Lastly, information and education was given to the study subjects with regard to malaria prevention, control, and early diagnosis and adequate treatment, and about HIV counselling and testing.

### Role of the funding source

The sponsors of the study had no role in study design, data collection, data analysis, data interpretation, or writing of the report. The corresponding author had full access to the data and had full responsibility for the decision to submit.

## Results

### Study demographics

A total of 810 adults provided data for analysis. The demographics of the study participants are reported in Table 1. Four hundred and fifty-seven individuals (56 %) reported having symptoms of suspected malaria lasting more than 2 days, and 75 (9 %) had sought health advice prior to the current presentation (Table 2). The overall prevalence of confirmed malaria was 25 % for participants in the study.

**Table 1:** Characteristics of participants

Characteristics	Total	Concern about covert HIV testing		
	(%) *N* = 810	Yes (%) *N* = 406	No (%) *N* = 404	*p* value*
Location				
Urban	476 (59)	230 (57)	246 (61)	0.22
Rural	334 (41)	176 (43)	158 (39)	
Sex				
Male	397 (49)	189 (47)	208 (51)	0.16
Female	413 (51)	217 (53)	196 (49)	
Age (years)				
16–24	313 (39)	159 (39)	154 (38)	0.09
25–34	299 (37)	155 (38)	144 (36)	
35–44	142 (18)	73 (18)	69 (17)	
44+	56 (7)	19 (5)	37 (9)	
Highest level of education				
Illiterate	209 (26)	119 (29)	90 (22)	0.15
Literate but no formal education	38 (5)	18 (4)	20 (5)	
Primary school	276 (34)	133 (33)	143 (35)	
Secondary school or higher	287 (35)	136 (33)	151 (37)	
Electricity in household				
Yes	529 (65)	255 (63)	274 (68)	0.13
No	281 (35)	151 (37)	130 (32)	

*Chi-squared test

**Table 2:** History of suspected malarial symptoms

Factors	Total	Location		*p* value*
		Urban	Rural	
Number of days of illness prior to health centre attendance (%)				
<1	78 (10)	53 (11)	25 (7)	0.02
1	62 (8)	38 (8)	24 (7)	
2	213 (26)	139 (29)	74 (22)	
3	251 (31)	140 (29)	111 (33)	
4+	206 (25)	106 (22)	100 (30)	
Sought health advice prior to visiting current health centre (%)				
Yes	75 (9)	34 (7)	41 (12)	0.01
No	735 (91)	442 (93)	293 (88)	
Took antimalarial tablets prior to attendance at health centre (%)				
Yes	65 (8)	23 (5)	42 (13)	>0.001
No	136 (17)	79 (17)	57 (17)	
No response	609 (75)	374 (79)	235 (70)	
Positive laboratory test for malaria (%)				
Yes	204 (25)	108 (23)	96 (29)	0.05
No	606 (75)	368 (77)	238 (71)	

*IQR* interquartile range

*Chi-squared test

### Concerns about HIV testing

Four-hundred and six (50 %) individuals considered that HIV testing was applied to blood given for malaria testing at the health centre (Table 3); 233 (29 %) of individuals considered that many or almost all members of the community had similar beliefs that malaria testing also involved HIV testing; 327 (40 %) of individuals considered that fear of HIV testing delayed presentation of individuals with suspected malaria. Participants who suspected that blood provided for malaria diagnosis was covertly tested for HIV had a median delay of three days (interquartile range IQR 2 to 4) from the start of their symptoms to clinical evaluation compared to a median of three days (IQR 2 to 3) for those who did not hold this belief (*p* < 0.01).

**Table 3:** Impact of health beliefs of participants on duration of symptoms

Factors	Total (%)	Median duration of symptoms, days (IQR)*	Duration of symptoms		*P* value*
			≤3 days	≥4 days	
Concerned about HIV testing when you visit a health facility for malaria diagnosis and treatment					
Yes	406 (50)	3 (2 to 4)	289 (48)	117 (57)	0.03
No	404 (50)	3 (2 to 3) *p* < 0.01	315 (52)	89 (43)	
How sure that covert HIV testing is done on malaria samples					
Do not believe	404 (50)	3 (2 to 3)	316 (52)	88 (43)	0.01
Maybe	212 (26)	3 (2 to 3.5)	159 (26)	53 (26)	
Very/completely sure	194 (24)	3 (2 to 4) *p* = 0.003	129 (21)	65 (32)	
Estimate of how many people in community believe that malaria blood samples are also tested for HIV					
None	269 (33)	2 (1 to 3)	219 (36)	50 (24)	0.004
Few	220 (27)	3 (2 to 3)	167 (28)	53 (26)	
Many	188 (23)	3 (2 to 4)	126 (21)	62 (30)	
Almost all/All	45 (6)	3 (2 to 4)	33 (5)	12 (6)	
Do not know	88 (11)	3 (2 to 4) *p* = 0.001	59 (10)	29 (14)	
Consider that the fear of HIV testing is the reason for the delay in people seeking an early diagnosis of malaria at health facility?					
Yes	327 (40)	3 (2 to 4)	223 (37)	104 (50)	<0.001
No	334 (41)	2 (2 to 3)	281 (47)	53 (26)	
Do not know	149 (18)	3 (2 to 4) *p* = 0.001	100 (17)	49 (24)	
Concerns about HIV testing are main reason for delay in presenting this time					
Yes	129 (16)	3 (2 to 3)	70 (12)	59 (29)	<0.001
No	681 (84)	3 (3 to 5) *p* < 0.001	534 (88)	147 (71)	

*IQR* interquartile range

*Non parametric test as appropriate (Mann–Whitney test, Kruskall-Wallis test)

Concerns about HIV testing on blood donated for the diagnosis of malaria were associated with delays in attending for treatment, with 117 individuals (57 %) of those with concerns waiting for 4 days or more, compared to 89 (43 %) who did not have any such concerns (*p* = 0.03). One hundred and twenty nine (16 %) individuals stated that concern about HIV testing was the main reason for the delay in seeking treatment, and 59 (46 %) of these individuals presented after more than three days of symptoms compared to 147 (22 %) of individuals who did not consider fear of HIV testing the cause for their delay (*p* < 0.001). Table 4 demonstrates that delays in seeking treatment varied between the five health centres (*p* < 0.001, chi-squared test). The stratified analysis suggested that much of the associations observed were driven by data from Meki health centre with an odds ratio for duration of symptoms greater than 3 days if patient had concerns about HIV testing of 8.72 (95 % confidence interval 3.63 to 20.97).

**Table 4:** Odds ratio of impact of health beliefs of participants on duration of symptoms stratified by health centre

Health centre	Total number of individuals	Number of individuals with symptoms ≥4 days (%)	Number of individuals concerned about HIV testing when you visit a health facility for malaria diagnosis and treatment (%)	Odds ratio of duration of symptoms ≥4 days if concerned about covert HIV testing (95 % confidence intervals)
Abosto	139	53 (38)	65 (47)	0.49 (0.24 to 0.98)
Batu	180	36 (20)	94 (52)	1.02 (0.49 to 2.14)
Bulbula	147	17 (12)	79 (54)	1.26 (0.45 to 3.52)
Meki	175	48 (27)	92 (53)	8.72 (3.63 to 20.97)
Mojo	169	52 (31)	76 (45)	1.67 (0.87 to 3.24)

## Discussion

This is the first quantitative study to specifically assess the prevalence of concerns about HIV testing and associations with the time to presentation at health centres of adults with suspected malaria living in Ethiopia. Our analysis shows that half of the individuals with suspected malaria were concerned that HIV testing was implemented on all blood sampled for the diagnosis of malaria, and that 40 % of our respondents considered that these beliefs will result in relatively later presentation at medical facilities for symptomatic patients. Collectively, these concerns about covert HIV testing were associated with delayed presentation for management of suspected malaria, with these associations a consequence of data from Meki health centre in particular.

The study has a number of strengths including the prospective assessment of a priori hypothesis from a population of adults with suspected malaria, who were unaware of the hypothesis being tested, with a very high response rate (minimising bias). The main limitation of these data is the issue of generalisabilty to other cultural settings. Although these observations are valid within the communities in Central Ethiopia that we studied, it is impossible to assume that these concerns regarding HIV testing and delayed access to health care in general are applicable elsewhere. In addition, our study population could be biased by severity of disease, so that those with more advanced disease are unable to attend the health centres, and hence our observations are pertinent only to those who attend health centres with symptoms of malaria, although this is unlikely to account for the association between delayed presentation and concerns about HIV testing. Our data should thus be considered to be hypothesis generating, and further regional studies are required in a number of different settings to determine how prevalent the beliefs are that covert HIV testing accompanies malaria blood testing in high HIV prevalence countries.

A delay of over two days before obtaining medical assessment after the initiation of symptoms of suspected malaria occurred in 56 % of our study population, and similar delays have been observed in Nigeria [8], Senegal [9], Ethiopia [10, 11] and India [12, 13]. However, our data were cross-sectional and demonstrating causality is not possible using this study design. Future studies should consider using representative prospective study designs to study local differences in baseline attitudes to HIV testing, and in particular their possible association with late presentation of patients with malarial symptoms, adjusting for potential confounding factors such as access to health facilities.

The current study was performed at the same time as a second study that tested a related hypothesis in children presenting with suspected malaria [7]. These data demonstrated that 51 % of guardians of these children perceived that HIV testing was done on blood donated for malaria diagnosis, which is similar to the 50 % reported in the current data. Children whose guardians were concerned about covert HIV testing in cases of suspected malaria presented later with a median delay of three days compared to those children whose guardians were not concerned about covert HIV testing who had a median value of two days. While antiretroviral testing and therapy is available in Ethiopia, the disease remains a public health concern with stigma of having HIV infection being a particular problem despite the regular promotion of health awareness campaigns to educate the local population. The analysis stratified by health centre demonstrated that the prevalence of concern about HIV testing was common across all the regions studied, with values ranging from 45 to 54 % of the populations studied, suggesting that this was not localised to just one area. However, only in one health centre (Meki) did this translate into a delay in presentation, and further investigation is required to account for this. The fact that level of education attained does not modify the concerns about covert HIV testing is an important negative finding, that should inform future investigations in this area, as these concerns are shared across all educational groups.

Our data can be compared with those from Gambella Region of Ethiopia in 2008, where the prevalence of refusal for HIV testing among women attending for antenatal care was 25 % [14], suggesting that there may be a substantial minority of individuals in Ethiopia who do not want to take advantage of HIV testing to know their serological status. To date, sparse data exist on how attitudes to HIV impact on presentation with symptoms of malaria. Individuals living in rural Cote d’Ivoire who were offered a rapid diagnostic testing assay for malaria reported that 67 % had concerns that the blood would be used for HIV testing [15]. In Uganda, two separate qualitative studies explored the feasibility of introducing rapid diagnostic malaria assays. In the first qualitative study, the local population again expressed concern that blood samples collected for malaria could be used for HIV testing rather than malaria [16]. In the second qualitative study, again individuals were worried that ‘in most communities people think that taking of blood means testing for HIV and some do not want their HIV status disclosed’ [17].

### Implications for policy

If concerns about HIV testing delay malaria diagnosis, then this is obviously a concern for the local authorities that provide public health services in these countries, as malaria is a common disease that infects approximately 200 million individuals annually [1] and it is well recognized that prompt treatment for those who present with the infection is important to reduce morbidity and mortality [18–20]. One option would be to evaluate further targeted education for the public [21, 22], so that awareness of the risk of delayed presentation with suspected malaria by the erroneous concern that covert HIV testing is administered to all blood tests is addressed directly. One other possible explanation for our data from this and our previous study in the same population [7] is that it is actually true, that at times healthcare workers test blood samples for HIV infection without fully informed consent of the patient. Future studies may wish to clarify this issue and that of the confidentiality of results in these communities, and could provide useful data to reassure the local population that their blood is only used for the purposes provided, which in itself may reduce some of the concerns observed in our study population.

## Conclusions

These data suggest that concerns about covert HIV testing without informed consent are common in central Ethiopia, and that these may result in delayed presentation by adults with suspected malaria for medical treatment. This provides a relatively novel risk factor that inhibits prompt treatment of patients with malarial symptoms that is potentially reversible, and provides the option of testing non-medical interventions to improve earlier presentation and hence treatment for this common infection.

## Abbreviations

HIV: human immunodeficiency virus
IQR: interquartile range
